# Enhancing ecological uncertainty predictions in pollution control games through dynamic Bayesian updating

**DOI:** 10.1038/s41598-024-63234-1

**Published:** 2024-06-01

**Authors:** Jiangjing Zhou, Ovanes Petrosian, Hongwei Gao

**Affiliations:** 1 Saint Petersburg State University, 7/9 Universitetskaya nab., St., Petersburg, 199034 Russia; 2 School of Automation, Qingdao University, Qingdao, 266071 China; 3 School of Mathematics and Statistics, Qingdao University, Qingdao, 266071 China

**Keywords:** Climate-change policy, Applied mathematics

## Abstract

This study presents a dynamic Bayesian game model designed to improve predictions of ecological uncertainties leading to natural disasters. It incorporates historical signal data on ecological indicators. Participants, acting as decision-makers, receive signals about an unknown parameter-observations of a random variable’s realization values before a specific time, offering insights into ecological uncertainties. The essence of the model lies in its dynamic Bayesian updating, where beliefs about unknown parameters are refined with each new signal, enhancing predictive accuracy. The main focus of our paper is to theoretically validate this approach, by presenting a number of theorems that prove its precision and efficiency in improving uncertainty estimations. Simulation results validate the model’s effectiveness in various scenarios, highlighting its role in refining natural disaster forecasts.

## Introduction

In the context of dynamic games, the progression of states is traditionally viewed as deterministic. The earliest literature on pollution control games typically assumed deterministic growth of pollution stocks^1^. However, this perspective fails to account for a significant aspect of real-world situations: the uncertainty surrounding model parameters. Players do not have complete knowledge of the unpredictability inherent in these parameters. Take, for example, the scientific community is still struggling to understand the ecological uncertainty of how pollution dissipates in oceans and forests, the specific impact of greenhouse gases on global warming, and the accurate prediction of future temperature changes. Additionally, there is uncertainty regarding the structure of the game itself. Players are often unaware of the exact nature of motion equations and payoff functions for the entire duration over which the game unfolds. As the game progresses, updates about the game’s structure are provided^2^.

The majority of papers addressing the occurrence of unknown parameters in game models can be found in key sources. The first introduction of uncertainty in pollution control problems was presented in^3^, where the author incorporated learning in a dynamic game of international pollution with ecological uncertainty. This study characterized and compared the non-cooperative emission strategies of players under conditions of unknown ecological uncertainty distribution, which was mitigated through learning. Paper^4^ explored resource extraction and investment games within a learning context. Paper^5^ examine a stochastic differential game in which the random variable representing consumers’ willingness to pay follows a uniform distribution. The alternative approach assumes that players lack precise knowledge of certain model parameters but hold beliefs about these unknowns based on available data. As new information is received, their beliefs are updated accordingly.

In this paper, we address a global environmental issue where neighboring nations release pollutants leading to accumulation that adversely affects the shared natural environment. We consider the accumulation process to be influenced by ecological uncertainty and permit the countries involved (as players) to gradually acquire knowledge about this uncertain parameter over time. Indeed, the player collects and analyzes data to learn about an unknown parameter using Bayesian updating approach. After receiving the data for each time period, the planner obtains an estimation of this unknown parameter and then selects the optimal control strategy based on this estimation. The payoff functions in this game are determined by the state, which players estimate based on their perceptions of an unknown parameter. These estimations guide the players in assuming how the game evolves, directly influencing their strategic decisions and the resulting payoffs based on these perceived states. We examine the impact of learning on the formulation of optimal policies.

On the other hand, players may lack information about changes in the game structure over the entire time interval, but possess certain insights about the game structure within a truncated time interval. The risk associated with structural uncertainty is alleviated through the application of dynamic updating methods^2^. The study^6^ delves into dynamic updating with stochastic forecasts and dynamic adaptation, particularly in scenarios where information about the conflicting process may evolve during the game. Additionally, dynamic updating has been applied to cooperative differential games^7–9^. Another method very similar to dynamic updating is continuous updating. In the works^10–13^, Nash equilibria in games with continuous updating are derived using Hamilton–Jacobi–Bellman equations and Pontryagin’s maximum principle. The realm of linear-quadratic differential games with continuous updating is explored in the studies^14,15^. These works delve into both cooperative and non-cooperative scenarios, providing corresponding solutions. Additionally, the research^16,17^ address cooperative differential games, further extending the applications of continuous updating in game theory.

Following the framework^18^, who explored a two-stage game with three distinct learning scenarios, namely, no learning (where players are unaware of stochastic parameter values before decision-making), partial learning (where players discover these values before making second-stage decisions), and full learning (where uncertainty is completely absent). We also propose three scenarios. These include the full information, where the distribution of the random variable is known, eliminating structural uncertainty; the dynamic updating, facing only structural uncertainty; and the learning, where both types of uncertainties are present. However, our exploration of these scenarios adopts a perspective distinctly different from those presented in previous studies^19,20^.

There are three main innovations in this paper:

1. The first significant innovation is the adaptation of Bayesian updating into a dynamic framework. This approach transforms Bayesian updating from a traditional single-stage process into a comprehensive multi-stage analysis. It enables an in-depth examination of the evolution of players’ beliefs across stages, where a player’s posterior belief at stage *t* seamlessly transitions to their prior belief at stage $t+1$.
2. Our second innovation departs from the conventional reliance on mathematical expectations to forecast the outcomes of random variables with indeterminate distributions at each stage. Instead, we employ conditional expectations based on players’ beliefs about the unknown parameters. This method shifts the focus from the inherent randomness of the variables to the players’ beliefs within the game’s context.
3. The third innovation extends the concept of dynamic updating to game models featuring unknown parameters. This novel approach maintains that players possess definitive knowledge of the game’s structure over a fixed period, as opposed to the entire duration of the game.

The remaining sections of the paper are structured as follows: Sect. 2 outlines three information scenarios for players, ranging from full awareness of the random variable’s distribution and game structure knowledge to acquiring such information through learning. Section 3 delves into Bayesian updating for normal distributions, comparing static and dynamic contexts. In Sect. 4, we explore a dynamic Bayesian updating model applied to pollution control, deriving a Nash equilibrium with dynamic Bayesian updating using the Hamilton-Jacobi-Bellman equation. Section 5 presents numerical simulation results to validate our theoretical framework. Finally, Sect. 6 concludes the paper by summarizing findings and suggesting avenues for further research.

## Information scenarios

This section introduces the foundational concepts by presenting three distinct scenarios in which players operate within games. Players’ knowledge of the random variable’s distribution and the structure of the game itself varies, ranging from complete awareness to the necessity of learning these elements through the game. For the reader’s convenience, we list key notations in Table 1.

**Table 1:** Notation Glossary.

$\Theta$	Sample space of the unknown parameters
$\theta$	Unknown parameter of the distribution of the random variable
${\overline{\theta }}_t$	Prior estimator of $\theta$ at stage *t*
$\xi _t(\theta )$	Belief distribution regarding $\theta$ held by players at stage *t*
$X_t$	Set of signals at stage *t*
$x_t$	The signal received at stage *t*

The dynamics of the system, in discrete time (spanning $t=t_0, t_1, ..., \infty$) for a game played by *N* players in a state space can be shown by the following state equation:

1 $$\begin{aligned} {S_{t+1}}=f( {{E({\widetilde{\eta }}}|\cdot )},{u_{1,t}},u_{2,t},...,u_{N,t},{S_t}), \quad {S_{t_0}=S_0}, \end{aligned}$$

where $\widetilde{\eta }$ is introduced to represent the randomness in the model’s parameters. Players lack foresight regarding the future realizations of the random variable before making a decision at each stage. In this case, $\widetilde{\eta }$ is a random variable whose value at each moment is unknown to the participants. Consider *x* to be a realization of the random variable $\widetilde{\eta }$, whose probability distribution is given by the function $\phi ({x}\vert \theta ^*)$, where $\theta ^*$, $\theta ^*\in \Theta \subset {R^l}$, is the vector of sufficient parameters of the probability density function (p.d.f.) $\phi$. In order to forecast the realization of the random variable ${\widetilde{\eta }}$ at time *t*, the player uses conditional expectation $E({\widetilde{\eta }}|\cdot )$ relies on their existing beliefs.

While the type of distribution that the random variable $\widetilde{\eta }$ follows is known, the parameters of that distribution, denoted as $\theta ^*$, remain unknown to the players. In this context, $\theta$ can be viewed as a random vector, denoted as $\theta = (\theta ^1, \ldots , \theta ^m)$, where each element is unknown and the parameters are related; that is, the players’ belief regarding an unknown parameter, indicated as $\theta ^1$, could be conditional on another parameter, denoted as $\theta ^2$.

**Figure 1:**
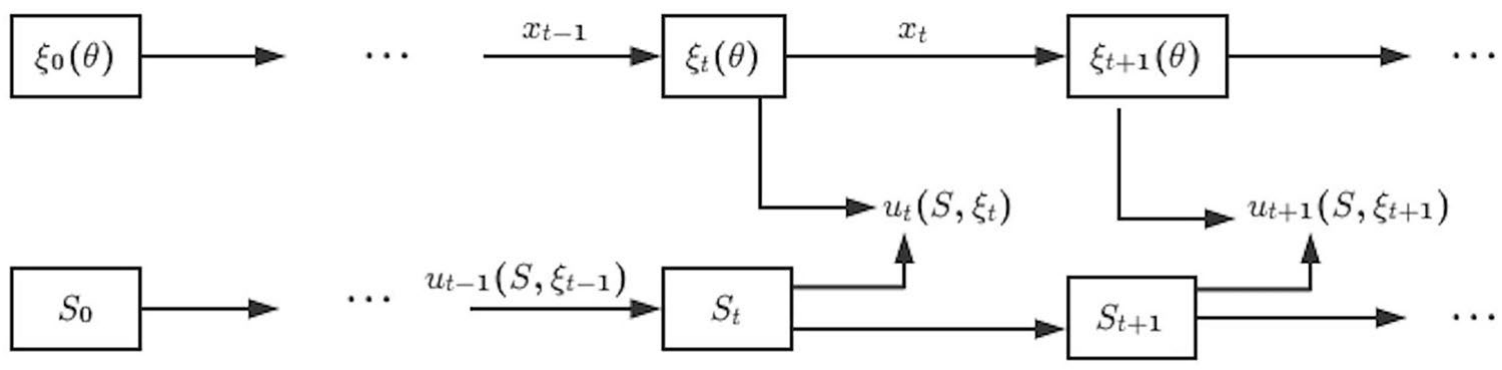
Game models with dynamic Bayesian updating information.

Figure 1 represents the game models with the dynamic Bayesian updating information structure for each player. However, players receive the realization of ${\widetilde{\eta }}$ at each time, allowing them to update their prior estimates of any unknown parameters. Consequently, at time *t*, players use the signal from time $t-1$ to derive their prior belief $\xi _t$ about the unknown parameter vector $\theta$, and based on this belief and the current system state *S*, they make decision $u_t(S, \xi _t)$. This process encapsulates the feedback loop between the evolving system state and the players’ decisions, underpinned by the adaptation of beliefs in response to new information about the stochastic elements of the model.

The payoff function for player *i* is presented below:

2 $$\begin{aligned} J_i(S_0, t_0; u_{t})=\sum _{t=t_0}^{+\infty }\rho ^tg^i[t,{u_{1,t}},u_{2,t},...,u_{N,t},S_t], \quad i=1,2,..., N, \end{aligned}$$

where the discount factor is $\rho \in (0, 1)$ and $u_t=(u_{1,t},u_{2,t},...,u_{N,t})$ is the strategy profile. In this context, the state $S_t$ at each stage is a deterministic value that is a function of the players’ control decisions and their beliefs regarding the unknown parameters. Therefore, when considering the players’ payoff functions, the system state at each stage becomes a known value when the players’ controls are fixed.

Three distinct scenarios are of primary interest in this model, each representing different levels of knowledge among the players:

1. Full Awareness Scenario: Players possess complete knowledge of both the distribution of random variables and the game’s structure throughout the entire stage. This scenario represents an ideal situation where players have comprehensive information.
2. Structural Uncertainty Scenario: Here, players are aware of the distribution of random variables. However, their understanding of the game’s structure is limited to a fixed number of stages rather than the entire duration of the game. This reflects a more realistic situation where players have incomplete but evolving knowledge of the game dynamics.
3. Learning Scenario: In this most complex scenario, players initially lack knowledge about both the distribution of random variables and the game’s structure. However, as the game progresses, they gradually acquire this information. This scenario mirrors real-world situations where players must adapt and learn based on their experiences and observations as the game unfolds.

### Incorporating structural uncertainties in game models

In this subsection, we delve into a scenario where the players are privy to the value of $\theta ^*$ but remain unaware of the complete equations of motion and payoff functions throughout the entire duration of the game, a situation that essentially models game structural uncertainty. To address this, we implement dynamic updating, which is particularly relevant when players have access to only partial game structure information, as opposed to comprehensive knowledge of the entire game structure.

At each time step *t*, players receive information pertinent to a set of upcoming stages, specifically $\{t, t+1,...,t+\overline{T}\}$, where $0< \overline{T}< +\infty$. This implies that while devising strategies at any given time *t*, players can only consider the information available for the interval from *t* to $t+\overline{T}$. In this context, our analysis focuses on the truncated subgame $\Gamma (S, t, t+\overline{T})$, examining how players strategize and make decisions based on the limited information available to them.

Furthermore, assume that the evolution of the state for the subgame $\Gamma (S, t, t+\overline{T})$ can be described by

3 $$\begin{aligned} S_{k+1}^t=f(\theta ^*,{u_{1,k}^t},u_{2,k}^t,...,u_{N,k}^t,{S_k^t}), \quad {S_{t}^t=S_t=S}, \quad k\in \{t, t+1,...,t+\overline{T}\}. \end{aligned}$$

Player *i*’s payoff function for the subgame $\Gamma (S, t, t+\overline{T})$ takes the following form:

4 $$\begin{aligned} J_i^t(S, t, t+\overline{T}; u_{k}^t)=\sum _{k=t}^{t+\overline{T}}\rho ^kg^i[k,{u_{1,k}^t},u_{2,k}^t,...,u_{N,k}^t,S_k^t], \end{aligned}$$

yielding optimal strategy ${\widetilde{u}}_{i,k}^{t,*}(S)$, $i=1,2,...,N$.

Strategy profile $u_k^t(S)$ in the games with dynamic updating has the form:

In the framework of dynamically updated information it is important to model the behavior of players. In order to accomplish this, we employ the notion of Nash equilibrium in our feedback strategies. However, in the case of games with dynamic updating, we prefer to have it in the following format: for any fixed $t\in \{t_0, t_1, ..., \infty \}, {\widetilde{u}}_{k}^{t,*}(S)=\left( \widetilde{u}_{1,k}^{t,*}(S), \ldots , {\widetilde{u}}_{N,k}^{t,*}(S)\right)$ coincides with the Nash equilibrium in the game (3), (4) defined on the interval $[t, t+\overline{T}]$ in the instant *t*.

Dynamic game with structural uncertainties is developed according to the following rule: Current time $t\in \{t_0, t_1,..., \infty \}$ evolves dynamically and as a result players dynamically obtain new information about motion equations and payoff functions in the game $\Gamma (x, t, t+\overline{T})$.

### Extending the incorporation of uncertainties in game models to include parameters

We now consider the scenario where the assumption of known $\theta ^*$ is relaxed. That is to consider the case of learning the unknown parameters as receiving the signals. Consequently, we aim to develop a mechanism that enables players to account for the uncertainty in model parameters during their decision-making process and to learn the values of these unknown parameters through experience.

Indeed, the learning planner makes decisions, anticipating updating beliefs every period. At the beginning of each stage, when the players have not received the current signal, the player needs to make decisions based on the past values of the signals. Upon observing the signal $x_t$ at stage *t*, players revise their beliefs and proceed to the subsequent stage.

When addressing parameter uncertainty, the primary emphasis is on how players utilize available information to methodically refine their views about the uncertain parameter. We first introduce the concept of Bayesian updating in a static context:

1. At the beginning of each stage $t$, players assign a prior belief $\xi _t(\theta )$ regarding the unknown parameter $\theta$. This belief is common knowledge among all players.
2. Upon reaching stage $t$, players observe the signal $x_t$, which contains information relevant to updating their beliefs about the unknown parameter $\theta$.
3. Using the Bayesian updating method, players update their beliefs dynamically to obtain the posterior belief ${\hat{\xi }}_{t}(\theta |x_t)$ based on the newly observed signal $x_t$.

Formally, given the prior belief $\xi _t(\theta )$ and signal $x_t$ at time *t*, the posterior belief $\hat{\xi _t}(\theta |x_t)$ is

5 $$\begin{aligned} \hat{\xi _t}(\theta |x_t)=\frac{\phi (x_t \vert \theta ) \xi _t(\theta )}{\int _{\Theta } \phi (x_t\vert y) \xi _t(y) \textrm{d} y}, \end{aligned}$$

sss for $\theta \in \Theta$, by Bayesian inference (5), characterizes the learning process through the updating of beliefs considering the information gleaned from observing $x_t$ at time *t*. Observing $x_t$ directly allows us to focus on the learning environment. Significantly, the learning process as characterized by (5) is independent of the control vector.

Following (5), we will articulate a pivotal assumption that bridges to the dynamic aspect of Bayesian updating, enabling a seamless transition from static to dynamic analysis of players’ belief updating processes. Consequently, we propose the following assumption:

#### Assumption 1

At each stage $t\in \{t_0, t_1, ..., \infty \}$, the posterior belief regarding the uncertain parameter $\theta$, ${\hat{\xi }}_{t}(\theta |x_t)$, serves as the prior belief for the next stage, $t+1$, formally expressed as $\xi _{t+1}(\theta ) = {\hat{\xi }}_{t}(\theta |x_t)$.

Assumption 1 establishes a linkage between consecutive stages, where the posterior of one stage becomes the prior for the next. Again, a new posterior can be obtained by updating the new prior with the likelihood generated from the new signal. This cycle can continue indefinitely; hence, our beliefs are continually revised.

In the context of game models with structural uncertainties, players navigate through the structural uncertainty by making decisions within defined information interval $\overline{T}$. At any instant $t$, the decision-making is strictly based on the information about game structure available within the interval $t$ to $t + \overline{T}$. As the game progresses to time $t+1$, a new information interval emerges, necessitating a fresh set of decisions based on the updated interval $t+1$ to $t+1 + \overline{T}$.

#### Assumption 2

In a game characterized by both structural uncertainties and parameter uncertainties, it is assumed that the players’ beliefs regarding unknown parameters within the information interval from $t$ to $t + \overline{T}$ remain unchanged at time $t\in \{t_0, t_1, ..., \infty \}$.

At each instant moment *t*, players operate within a specific information interval concerning the game’s structure. Coupled with Assumption 2, this framework posits that within this interval, players’ beliefs about the model’s unknown parameters remain unchanged due to the absence of new signals. Consequently, throughout the subgame defined by this interval, there is essentially no update to the information upon which decisions are based. Therefore, the unknown parameters of the model are considered to be constant and deterministic values, anchored to the players’ current beliefs. Players then use the dynamics and payoff functions defined for this interval to select their optimal strategies.

Consider N-player subgame $\Gamma (S,t,t+\overline{T})$, $t\in \{t_0,...,+\infty \}$, defined on the discrete time instances $\{t, t+1,...,t+\overline{T}\}$, where $0<\overline{T}<+\infty$.

The equations of motion for the subgame $\Gamma (S,t,t+\overline{T})$ for each fixed time *t* has the form:

6 $$\begin{aligned} S_{k+1}^t&=f({E(\widetilde{\eta }\vert {\overline{\theta }}_t)},{u_{1,k}^t},u_{2,k}^t,...,u_{N,k}^t,{S_k^t}), \quad {S_{t}^t=S_t=S},\\ {\overline{\theta }}^i_t&=\int _{\Theta ^i} \theta ^i\xi _t(\theta ^i)d\theta ^i,\quad i=1,2,...,m, \end{aligned}$$

where $\xi _t(\theta ^i)$ denotes the prior belief concerning the unknown parameter $\theta ^i$ at stage $t$. $\overline{\theta }_t^i$ represents the estimated value of the unknown parameter $\theta ^i$ at time $t$. This estimation is obtained by calculating the mathematical expectation using the player’s prior opinion. Subsequently, $E(\widetilde{\eta } \vert \overline{\theta }_t)$ represents the estimated value of the random variable ${\widetilde{\eta }}$ using conditional mathematical expectation. It is evident that Eq. (6) is influenced by the variable *S* and the prior probability density function (p.d.f.) $\xi$ on $\Theta$. Indeed, the state space is $(S, \xi )$.

Payoff function of player *i* for the subgame $\Gamma (S,t,t+\overline{T})$ is described by (4).

In order to construct such strategies in the subgame, we first consider the concept of generalized Nash equilibrium with dynamic Bayesian updating:

#### Definition 1

Strategies $\{\widetilde{u}_{i,k}^{t,*}(S,\xi _t(\theta ))\}_{i=1,2,..., N}$ are generalized Nash Equilibrium strategy with dynamic Bayesian updating (generalized NEDBU) of the game $\Gamma (S,t,t+\overline{T})$, if for any fixed $t\in \{t_0,t_1,...,+\infty \}$, strategy profile $\widetilde{u}_{k}^{t,*}(S,\xi _t(\theta ))=(\widetilde{u}_{1,k}^{t,*}(S,\xi _t(\theta )),..., {\widetilde{u}}_{N, k}^{t,*}(S,\xi _t(\theta )))$ is the feedback Nash equilibrium in game $\Gamma (S,t,t+\overline{T})$.

In this article, the Hamilton-Jacobi-Bellman Equations will be used to determine the generalized NEDBU of players.

#### Theorem 1

For each fixed time $t\in \{t_0,t_1,...,+\infty \}$, a set of strategies $\{\widetilde{u}_{i,k}^{t,*}(S,\xi _t(\theta ))\}_{i=1,2,..., N}$ provides a generalized NEDBU, if there exist functions $V_i^t(k, S,\xi _t(\theta )), i=1,2,...,N$, for $k=t,t+1,...,t+\overline{T}$, such that the following recursive relations are satisfied:

7 $$\begin{aligned}&V_i^t(k, S,\xi _t(\theta ))= \max \limits _{u_{i,k}^t} \left\{ \rho ^k g^i[k,S,{u_{1,k}^t},u_{2,k}^t,...,u_{N,k}^t]+V_i^t\left( k+1,f({E(\widetilde{\eta }\vert {\overline{\theta }}_t)},{u_{1,k}^t},u_{2,k}^t,...,u_{N,k}^t,{S}),\xi _t(\theta )\right) \right\} ,\\&V_i^t(t+\overline{T}+1, S,\xi _t(\theta ))=0. \end{aligned}$$

#### Proof

The detailed proof is provided in the “Supplementary Materials”. $\square$

By using a strategy based on generalized NEDBU, found within each specific subgame at every time point *t*, we establish a solution for the entire game. This strategy, specific to subgames at each instant, helps define a consistent approach for players’ decisions across the whole game timeline.

#### Definition 2

The definition for the Nash Equilibrium strategy with dynamic Bayesian updating (NEDBU) is as follows:

8 $$\begin{aligned} u_{i,t}^*(S,\xi _t(\theta ))=\widetilde{u}_{i,k}^{t,*}(S,\xi _t(\theta ))\vert _{k=t},\quad t\in \{t_0,t_1,...,+\infty \}, i=1,2,...,N, \end{aligned}$$

where ${\widetilde{u}}_{i,k}^{t,*}(S,\xi _t(\theta ))$ is the generalized NEDBU which we defined in Definition 1.

After selecting a strategy at time $t$, players receive a signal $x_t$ about the unknown parameter at that instant. This signal enables players to update their beliefs about the unknown parameter to ${\hat{\xi }}_t(\theta )$. Utilizing Assumption 1, we infer that the prior belief about the unknown parameter at time $t+1$, denoted as $\xi _{t+1}(\theta )$, is established. According to Assumption 2, this belief remains unchanged over the interval from $t+1$ to $t+1 + \overline{T}$.

At time $t+1$, players are informed about the game structure defined within the new information interval $\overline{T}$, encompassing dynamics and payoff functions from $t+1$ to $t+1 + \overline{T}$. Armed with this information and the system state $S_{t+1}$, players determine the optimal strategy for this interval.

If it is feasible to derive a generalized NEDBU, denoted as ${\widetilde{u}}_{i,k}^{t,*}(S,\xi _t(\theta ))$, through the application of Eq. (7), then the procedure outlined in (8) can be employed to acquire the strategy profile, $u_{i,t}^*(S,\xi _t(\theta ))$. This strategy profile, $u_{i,t}^*(S,\xi _t(\theta ))$, will subsequently serve as the foundational solution concept in the dynamic game framework, which is characterized by both parameter uncertainty and structural uncertainty.

## Bayesian updating for estimating unknown parameters in normal distributions

Initially, we investigate the situation in which both the mean and precision of a distribution need to be acquired. Let’s examine ${\widetilde{\eta }}$, which is a random variable that has a normal distribution with unknown parameters $\theta ' = (\mu , \sigma ^2)$. Our research primarily focuses on the precision of the normal distribution, which is defined as the inverse of the variance ($\lambda = \frac{1}{\sigma ^2} > 0$). The objective is to estimate the parameters $\mu$ and $\lambda$ using Bayesian methods, resulting in the expression $\theta =(\mu , \lambda )$.

Before we delve into the Bayesian updating process, it is essential to elucidate the transition from the players’ joint beliefs about unknown parameters to their beliefs regarding each distinct parameter, facilitated through the application of marginal probabilities.

At stage *t*, $\xi _t(\theta )$ represents the joint prior belief of $\mu$ and $\lambda$. To ascertain the players’ beliefs about each separate parameter, we utilize the marginal distribution method, expressed as follows:

9 $$\begin{aligned} \xi _t(\mu )=\int _{0}^{\infty } \xi _t(\mu , \lambda ) d \lambda . \end{aligned}$$

Accordingly, we propose estimating the values of $\mu$ at each stage based on their mathematical expectations within the distribution. Let

$$\begin{aligned} {\overline{\mu }}_{t}=\int _{-\infty }^{\infty }\mu \xi _{t}(\mu )d\mu , \end{aligned}$$

denote the estimation of $\mu$ at the beginning of the stage *t*.

### Static Bayesian approaches

Subsequently, we will establish the initial moment of the game, denoted as $t_0$, to be zero. Our next objective is to compute the posterior distribution of the unknown parameters of the normal distribution using Bayesian inference. This will allow us to update our beliefs from the starting stage 0 to stage 1 in a single step.

**Prior belief for unknown precision** $\lambda$ **and unknown mean **$\mu$: The player’s prior belief for the unknown parameter vector $\theta =(\mu , \lambda )$ is assumed to follow a conjugate normal-Gamma distribution^21^:

$$\begin{aligned} \xi _0(\theta )&{\mathop {=}\limits ^{ \text{ def } }} \mathcal {N}\left( \mu \vert \mu _{0},\left( \kappa _{0} \lambda \right) ^{-1}\right) \times G a\left( \lambda \vert \alpha _{0}, \text{ rate } =\beta _{0}\right) \\&=\frac{1}{Z_{N G}\left( \mu _{0}, \kappa _{0}, \alpha _{0}, \beta _{0}\right) } \lambda ^{\frac{1}{2}} \exp \left( -\frac{\kappa _{0} \lambda }{2}\left( \mu -\mu _{0}\right) ^{2}\right) \lambda ^{\alpha _{0}-1} e^{-\lambda \beta _{0}} \\ {}&=\frac{1}{Z_{N G}} \lambda ^{\alpha _{0}-\frac{1}{2}} \exp \left( -\frac{\lambda }{2}\left[ \kappa _{0}\left( \mu -\mu _{0}\right) ^{2}+2 \beta _{0}\right] \right) , \end{aligned}$$

where $Z_{N G}\left( \mu _{0}, \kappa _{0}, \alpha _{0}, \beta _{0}\right) =\frac{\Gamma \left( \alpha _{0}\right) }{\beta _{0}^{\alpha _{0}}}\left( \frac{2 \pi }{\kappa _{0}}\right) ^{\frac{1}{2}}$, $``Ga''$ denotes the gamma distribution, while “$\mathcal {N}$” represents the normal distribution. Additionally, the prior belief of $\mu$ is explicitly conditional on $\lambda$. The parameter $\kappa _{0}$ is referred to as the prior’s equivalent sample size. This implies that when $\lambda =\frac{1}{\sigma ^2}$ is small, the variance of $\mu$’s prior is similarly significant.

The players’ prior belief in unknown parameter $\mu$ can be computed using Eq. (9) in the following manner:

10 $$\begin{aligned} \xi _0(\mu )&=\int _{0}^{\infty } \xi _0\left( \mu , \lambda \right) d \lambda \\&\propto \int _{0}^{\infty } \lambda ^{\alpha _{0}+\frac{1}{2}-1} \exp \left( -\lambda \left( \beta _{0}+\frac{\kappa _{0}\left( \mu -\mu _{0}\right) ^{2}}{2}\right) \right) d \lambda . \end{aligned}$$

We identify (10) as an unnormalized $G a\left( a, \text{ rate } =b\right)$ distribution, where $a=\alpha _{0}+\frac{1}{2}$ and $b=\beta _{0}+\frac{\kappa _{0}\left( \mu -\mu _{0}\right) ^{2}}{2}$. Consequently, we can express it simply as

11 $$\begin{aligned} \xi _0(\mu )&\propto \frac{\Gamma (a)}{b^a}\\&\propto b^{-a}\\&=\left( \beta _{0}+\frac{\kappa _{0}}{2}\left( \mu -\mu _{0}\right) ^{2}\right) ^{-\alpha _{0}-\frac{1}{2}} \\&\propto \left( 1+\frac{1}{2 \alpha _{0}} \frac{\alpha _{0} \kappa _{0}\left( \mu -\mu _{0}\right) ^{2}}{\beta _{0}}\right) ^{-\left( 2 \alpha _{0}+1\right) / 2}. \end{aligned}$$

At the initial stage $t = 0$, the player’s prior belief regarding the unknown parameter $\mu$ is quantified through (11). This belief is recognized as a location-scale *t*-distribution, denoted as $T_{2 \alpha _{0}}\left( \mu _{0}, \frac{\beta _{0}}{\alpha _{0} \kappa _{0}}\right)$, where $\mu _{0}>0$ is the mean, $2\alpha _{0}$ is the degree of freedom, and $\beta _{0} /(\alpha _{0} \kappa _{0})>0$ is the scale. It is imperative to note that this distribution embodies the player’s belief at stage $t=0$ concerning the parameter $\mu$, and is contingent upon the belief parameters $\mu _0, \kappa _0, \alpha _0,$ and $\beta _0$.

For those who are not familiar with the location-scale t-distribution, we provide the following brief explanation. Assuming that the degrees of freedom, $\nu$, are greater than 2, the *t*-distribution in the location-scale format, denoted as $T_{\nu }(\mu , \sigma ^2)$, where $\mu$ and $\sigma ^2$ represent the location and scale parameters respectively, exhibits these primary characteristics: The distribution’s mean is $\mu$, reflecting the central location around which the data are dispersed. The variance is $\frac{\nu }{\nu - 2} \sigma ^2$, indicating how the data spreads around the mean.

Note that $\alpha _0$ is set to $\frac{3v}{2}$, where *v* is any positive integer, ensuring that the degrees of freedom are always positive integers and the variance is positive at any given time.

The player’s Bayesian estimator of the unknown parameter $\mu$ at stage 0 is obtained using the following derivation:

$$\begin{aligned} {\overline{\mu }}_0=\int _{-\infty }^{\infty } \mu \xi _0(\mu )d\mu =\mu _0. \end{aligned}$$

The player’s prior belief about the unknown parameter $\lambda$ at stage 0 can be obtained in a similar manner:

12 $$\begin{aligned} \xi _0(\lambda )&=\int _{-\infty }^{\infty } \xi _0\left( \mu , \lambda \right) d \mu \\&= \frac{\beta _0^{\alpha _0}}{\Gamma (\alpha _0)} \lambda ^{\alpha _0-1} \exp (-\lambda \beta _0)\int _{-\infty }^{\infty } \frac{(\kappa _{0}\lambda )^{\frac{1}{2}}}{\sqrt{2\pi }}\exp \left( {-\frac{\kappa _{0}\lambda (\mu -\mu _0)^2}{2}}\right) d\mu \\&=\frac{\beta _0^{\alpha _0}}{\Gamma (\alpha _0)} \lambda ^{\alpha _0-1} \exp (-\lambda \beta _0). \end{aligned}$$

In the second line of Eq. (12), there is a normal distribution denoted as $\mathcal {N}(\mu _0, (\kappa _{0} \lambda )^{-1})$. The third line of Eq. (12) can be obtained from the property of the probability density function.

The player’s prior belief regarding the unknown parameter $\lambda$ at the initial stage $t = 0$ is conceptualized as a Gamma distribution. Specifically, this belief is represented mathematically by stating that $\lambda$ follows a Gamma distribution with parameters $\alpha _0$ and $\beta _0$, denoted as $\text {Ga}(\alpha _0, \beta _0)$. This distributional assumption is grounded in the findings from Eq. (12), which delineates the deduced prior belief for $\lambda$ based on initial belief parameters $\alpha _0, \beta _0$.

**The likelihood function:** Next, we will provide an overview of the typical knowledge and learning mechanisms that are accessible to players. Although the exact value of $\theta ^*$ is not known, the players have a prior belief that can be described by a normal-gamma prior probability density function on $\Theta$. It is important to mention again that these signals represent the realization of the random variable $\widetilde{\eta }$ at a given time, offering a current understanding of the unknown parameters. Since all players receive the same signals, these signals jointly contribute to a common belief.

Assume that players observe the signal follows a normal distribution with mean $\mu$ and precision $\lambda$, which can be performed as

$$\begin{aligned} \phi (x_0 \vert \mu , \lambda ) =\frac{1}{(2 \pi )^{1 / 2}} \lambda ^{1 / 2} \exp \left( -\frac{\lambda }{2} \left( x_0-\mu \right) ^{2}\right) . \end{aligned}$$

**Posterior belief for unknown mean** $\mu$ **and unknown precision** $\lambda$: After observing the signal $x_0$, the posterior distribution of the parameter $\theta$ is derived by updating the prior distribution with the likelihood function. Specifically, the signal $x_0$ represents the realization of the random variable ${\widetilde{\eta }}$ at the time $t = 0$. This process of updating encapsulates the incorporation of new evidence obtained through the observation of $x_0$, thereby allowing for a refined inference regarding the unknown parameter vector$\theta$:

$$\begin{aligned} \hat{\xi }_0(\theta )&\propto \phi (x_0\vert \mu ,\lambda ) \xi _0\left( \theta \right) \\&\propto \quad \lambda ^{\frac{1}{2}} e^{-\left( \kappa _{0} \lambda \left( \mu -\mu _0\right) ^{2}\right) / 2} \lambda ^{\alpha _0-1} e^{-\beta _0 \lambda } \times \lambda ^{1 / 2} e^{-\frac{\lambda }{2} \left( x_0-\mu \right) ^{2}} \\&\propto \quad \lambda ^{\frac{1}{2}} \lambda ^{\alpha _0+1 / 2-1} e^{-\beta _0 \lambda } e^{-(\lambda / 2) [\kappa _{0}(\mu -\mu _0)^{2}+(x_0-\mu )^{2}]}. \end{aligned}$$

It can be shown that

$$\begin{aligned} \kappa _{0}\left( \mu -\mu _0\right) ^{2}+\left( x_0-\mu \right) ^{2}&=\kappa _{0}\left( \mu -\mu _0\right) ^{2}+(\mu -x_0)^{2} \\&=\left( \kappa _{0}+1\right) \left( \mu -\mu _1\right) ^{2}+\frac{\kappa _{0} \left( {x_0}-\mu _0\right) ^{2}}{\kappa _{0}+1}, \end{aligned}$$

where $$\mu _1=\frac{\kappa _{0}\mu _0+x_0}{\kappa _{0}+1}$$.

Through calculation, we can obtain:

13 $$\begin{aligned} \hat{\xi }_0(\theta ) \propto \lambda ^{\frac{1}{2}} e^{-(\lambda / 2)(\kappa _0+1)\left( \mu -\mu _1\right) ^{2}} \times \lambda ^{\alpha _0+1 / 2-1}e^{-\beta _0\lambda } e^{-(\lambda / 2) \frac{\kappa _{0}(x_0-\mu _0)^2}{\kappa _{0}+1}}. \end{aligned}$$

By incorporating constants that do not alter Eq. (13), thereby facilitating the posterior belief to align with a canonical probability distribution, we can derive the subsequent posterior distribution:

$$\begin{aligned} \hat{\xi }_0(\theta )=\mathcal {N}\left( \mu \vert \frac{\kappa _{0}\mu _0+x_0}{\kappa _{0}+1},((\kappa _0+1) \lambda )^{-1}\right) \times G a\left( \lambda \vert \alpha _0+\frac{1}{2}, \beta _{0}+\frac{\kappa _{0} \left( {x_0}-\mu _0\right) ^{2}}{2\left( \kappa _{0}+1\right) }\right) . \end{aligned}$$

This exemplifies the static Bayesian updating process, wherein the player updates their beliefs about an unknown parameter vector to the posterior beliefs after receiving the signal $x_0$. Specifically, this static update is not a continuous process; in static Bayesian updating, each step begins anew without dependence on previous updates. We will next describe the methodology for dynamic Bayesian updating. Unlike the static approach, dynamic Bayesian updating maintains a connection between successive updates, making it a continuous process.

### Dynamic Bayesian approaches

At the start of each stage $t=0,1,2,...,\infty$, players possess normal-gamma conjugate prior beliefs regarding the unknown parameter vector $\theta$. These beliefs are specified as follows:

$$\begin{aligned} \xi _t(\theta ) {\mathop {=}\limits ^{{\phantom{0}}}} \mathcal {N}\left( \mu \vert \mu _{t},\left( \kappa _{t} \lambda \right) ^{-1}\right) \times G a\left( \lambda \vert \alpha _{t}, \text{ rate } =\beta _{t}\right) . \end{aligned}$$

Based on the marginal probability formula from (9), the player’s prior belief at time *t* regarding the unknown parameter $\mu$ as

$$\begin{aligned} \xi _t(\mu )&=\int _{0}^{\infty } \xi _t\left( \mu , \lambda \right) d \lambda ,\\&\propto \left( 1+\frac{1}{2 \alpha _{t}} \frac{\alpha _{t} \kappa _{t}\left( \mu -\mu _{t}\right) ^{2}}{\beta _{t}}\right) ^{-\left( 2 \alpha _{t}+1\right) / 2}, \end{aligned}$$

which is recognized as a location-scale *t*-distribution, denoted as $T_{2 \alpha _{t}}\left( \mu _{t}, \beta _{t} /\left( \alpha _{t} \kappa _{t}\right) \right)$. Here, $\mu _{t}>0$ represents the mean, $2\alpha _{t}$ represents the degree of freedom, and $\beta _{t} /(\alpha _{t} \kappa _{t})>0$ is the scale.

The player utilizes the conditional mathematical expectation, taking into account their priors belief about the unknown parameter, to estimate the value of $\mu$ at stage *t*.

$$\begin{aligned} {\overline{\mu }}_t=\int _{-\infty }^{\infty } \mu \xi _t(\mu )d\mu =\mu _t, \end{aligned}$$

where $\overline{\mu }_t$ demonstrates the player’s estimation of the unknown parameter $\mu$ at time $t$, equals the average of player’s belief $\xi _t(\mu )$ about $\mu$.

The variance of the player’s prior belief regarding the unknown parameter can be easily derived from the t-distribution produced earlier, which is given by

14 $$\begin{aligned} \frac{2 \alpha _{t}}{2 \alpha _{t}-2}\beta _{t} /\left( \alpha _{t} \kappa _{t}\right) =\frac{\beta _{t}}{\kappa _{t}\left( \alpha _{t}-1\right) }, \end{aligned}$$

represents the level of uncertainty in the player’s belief on the unknown parameter $\mu$.

Using Bayesian inference (5), player’s posterior belief of unknown parameter vector $\theta$ can be determined as follows:

$$\begin{aligned} \hat{\xi }_t(\theta )=&\phi (x_t\vert \mu ,\lambda )\xi _t(\theta ) \\ \propto&\mathcal {N}\left( \mu \vert \frac{\kappa _{t}\mu _t+x_t}{\kappa _{t}+1},((\kappa _t+1) \lambda )^{-1}\right) \times G a\left( \lambda \vert \alpha _t+\frac{1}{2}, \beta _{t}+\frac{\kappa _{t} \left( {x_t}-\mu _t\right) ^{2}}{2\left( \kappa _{t}+1\right) }\right) . \end{aligned}$$

By utilizing Assumption 1 and leveraging the fact that the distribution is conjugate, we deduce the subsequent formula for belief parameters:

15 $$\begin{aligned} \mu _{t+1}&=\frac{\kappa _{t} \mu _{t}+ x_{t}}{\kappa _{t}+1}, \\ \kappa _{t+1}&=\kappa _{t}+1, \\ \alpha _{t+1}&=\alpha _{t}+\frac{1}{2}, \\ \beta _{t+1}&=\beta _{t}+\frac{\kappa _{t} (x_{t}-\mu _{t})^{2}}{2(\kappa _{t}+1)}, \end{aligned}$$

where stage $t$ ranges over $\{0,1,2,...,\infty \}$. It is determined by the belief parameters $\mu _0, \kappa _{0}, \alpha _0$, and $\beta _0$, which reflect the player’s initial prior belief at stage 0.

After initially presenting the iterative Eq. (15), it is imperative to delve into its expanded form to lay the groundwork for subsequent proofs.

16 $$\begin{aligned} \mu _{n}&=\frac{1}{\kappa _{0}+n}\left( \kappa _{0} \mu _{0}+x_{0}+x_{1}+x_{2}+\cdots +x_{n-1}\right) ,\\ \kappa _{n}&=\kappa _{0}+n,\\ \alpha _{n}&=\alpha _{0}+\frac{n}{2},\\ \beta _{n}&=\beta _{0}+\sum _{m=0}^{n-1} y_{m}, \quad where\quad y_{m}=\frac{\kappa _{m}\left( x_{m}-\mu _{m}\right) ^{2}}{2 \cdot (\kappa _{m}+1)}, \end{aligned}$$

where $n=1,2,...,\infty$. The estimation of $\mu _n$ is calculated using a specific weighted averaging method that takes into account the initial estimate $\mu _0$, initial weight $\kappa _0$, and a series of observed values $x_0, x_1, \ldots , x_n$. Consequently, Eq. (16) elucidates how the player’s estimation evolves over time by accumulating signals received up to the previous moment.

In a more generalized scenario, when the observed values are regarded as random variables, indicated by $X_n$, with $n=0,1,2...,\infty$, the corresponding $\mu _n$ is also considered as a random variable, represented as $M_n$. Consequently, we assign $M_n$, $B_n$, and $Y_n$ as the corresponding random variables for $\mu _n$, $\beta _n$, and $y_n$, respectively.

The expected value of $M_n$, denoted by $E(M_n)$, represents the average performance of $M_n$ under various sample conditions. As the number of observations, represented by $n$, increases, the expected value $E(M_n)$ gradually converges towards the true mean $\mu$.

#### Theorem 2

   The expected estimation of the unknown mean will tend to the real value, which means

$$\begin{aligned} \lim _{n \rightarrow \infty } E(M_n)=\mu , \end{aligned}$$

where $M_n$ is the random variable describing the related belief parameter $\mu _n$ at stage *n*; it is the belief parameter of the unknown mean $\mu$ at stage *n*.

#### Proof

The detailed proof is provided in the “Supplementary Materials”.

$$\square$$

Next, we analyze the average performance of the variance between the estimated mean and the true value, as calculated in Eq. (14). Through this analysis, our objective is to demonstrate that the expected value of $\frac{B_n}{\kappa _n(\alpha _n - 1)}$ is anticipated to converge towards zero. This convergence underscores the reliability of our estimation method across various sampling contexts.

#### Theorem 3

   The expected variance of the estimation of the unknown mean tends to 0. In other words,

$$\begin{aligned} \lim _{n \rightarrow \infty } E\left( \frac{B_{n}}{\kappa _{n}\left( \alpha _{n}-1\right) }\right) =0, \end{aligned}$$

where $\frac{B_{n}}{\kappa _{n}(\alpha _{n}-1)}$ is a random variable representing the variance of the estimator of the unknown mean at the stage *n*, whereas $\kappa _{n}$, $\alpha _n$ are the belief parameters at the stage *n*.

#### Proof

The detailed proof is provided in the “Supplementary Materials”. $\square$

In summary, this predictive approach underscores the dynamic aspect of Bayesian updating. By dynamically updating the estimates and predictions based on the evolving data, the model adapts to new information, enhancing the accuracy and reliability of the predictions over time.

## Integrating parameter and structural uncertainty in pollution control models

The following example serves to demonstrate the practicality of our technique in addressing unknown distribution parameters and unknown game structures. We begin by introducing the optimal strategies of a learning planner, subsequently followed by the strategies of full awareness and the scenario of structural uncertainty.

Given the uncertainty in the game structure and that players are aware of information about the game structure from time *t* to $t+\overline{T}$, we construct the subgame $\Gamma (S,t,t+\overline{T})$ at each instant $t \in \{0,1,2,...,\infty \}$. It is important to note that in this definition of the subgame, it is assumed players do not receive new signals, hence their estimates of unknown parameters remain unchanged within this time frame. Consequently, we can state that neither parameter uncertainty nor structural uncertainty exists during this period.

For each given $t \in \{0,1,2,...,\infty \}$, the equations of motion for the subgame $\Gamma (S,t,t+\overline{T})$ are as follows:

17 $$\begin{aligned} S_{k+1}^{t}= \overline{\eta }_t\left( \sum _{i=1}^{N} u_{i,k}^t + \delta S_k^t\right) ,\quad {S_t^t = S_t = S}, \quad k \in \{t,t+1,...,t+\overline{T}\}, \end{aligned}$$

where $0< 1 - \delta < 1$ is the natural decay rate of pollution. The function $\overline{\eta }_t$ is defined as $E({\widetilde{\eta }}\vert \mu _t, \alpha _t/\beta _t)$. The predicted value of ${\widetilde{\eta }}$ is determined by $\mu _t, \kappa _t, \alpha _t$, and $\beta _t$ at stage *t*. The iterative Bayesian updating process is defined as (15). The state variable at stage $k+1$ is denoted as $S_k+1$. The variable $u_{i,k}^t$ represents the strategy of player *i* at stage *k*.

Consider $N$ nations or players, each producing a quantity $q_{i,t}$ of goods at time $t = 0, 1, ..., \infty$, indexed by $i = 1, ..., N$. Each unit of production results in an equivalent unit of pollution, $u_{i,t}$. The revenue for nation $i$ at time $t$, $z_{i,t}$, is a quadratic function of its emissions:

$$\begin{aligned} z_{i,t} = u_{i,t}(a_i - u_{i,t} - \gamma \sum _{j \ne i}^{N} u_{j,t}), \end{aligned}$$

where $a_i$ and $\gamma$ are positive constants for $i = 1,2,...,N$.

Each nation aims to balance its revenue with environmental costs, approximated by $D_i(S_t) = b_iS_t$ for $i = 1,2,...,N$, where $b_i>0$ is the marginal cost of the pollution stock. For each fixed $t \in \{0,1,2,...,\infty \}$, the payoff function for player $i$ in the subgame $\Gamma (S,t,t+\overline{T})$ is formulated as follows:

18 $$\begin{aligned} \max \limits _{u_{i,k}^t} J_i^t(S, t, t+\overline{T}; u_{k}^t) = \max \limits _{u_{i,k}^t}\sum _{k=t}^{t+\overline{T}}\rho ^k\left( u_{i,k}^t(a_i - u_{i,k}^t - \gamma \sum _{j \ne i}^{N} u_{j,k}^t) - b_iS_k^t\right) , \end{aligned}$$

where $S_k^t$ is constrained from the (17).

Our objective is to derive Nash equilibrium strategies with dynamic Bayesian updating across the entire span of the game, defined from $t = 0$ to $t = \infty$.

The methodology for constructing the Nash Equilibrium with Dynamic Bayesian Updating (NEDBU) is delineated as follows:

For each discrete time $t\in \{0, 1, 2, \ldots , \infty \}$, a corresponding truncated subgame $\Gamma (S, t, t + \overline{T})$ is identified, where $\overline{T}$ denotes the information horizon. By resolving the truncated subgame, which is formulated by the dynamic Eq. (17) in conjunction with the payoff function (18), we ascertain the generalized NEDBU (${\widetilde{u}}_{i,k}^{t,*}(\cdot )$, $k \in \{t,t+1,...,t+\overline{T}\}$, $i\in \{1,2,..., N\}$). Subsequently, through the application of the procedure delineated in (8), the NEDBU pertinent to time $t$ is derived.

### Proposition 4

The NEDBU for the game model, which is defined for the entire game, $t=0,1,2,...,\infty$, based on the solution of each truncated subgame and utilizing procedure (8), can be derived as follows:

19 $$\begin{aligned} u_{i,t}^*(S,\mu _t, k_t, \alpha _t, \beta _t)=&\frac{a_i}{2-\gamma }-\frac{\gamma a}{(2-\gamma +\gamma N)(2-\gamma )}\\&+\frac{{\overline{\eta }}_t}{(2-\gamma )(1-{\overline{\eta }}_t\delta \rho )}\left( b_i-\frac{\gamma b}{2-\gamma +\gamma N}\right) \left( (\overline{\eta }_t\delta )^{\overline{T}}\rho ^{\overline{T}+1}-\rho \right) , \\&\quad \text {for } i=1,2,...,N, \end{aligned}$$

where $${\overline{\eta }}_t=E({\widetilde{\eta }}\vert \mu _t, \alpha _t/\beta _t)$$, $$a=\sum _{i=1}^N a_i$$, and $$b=\sum _{i=1}^N b_i$$.

### Proof

The detailed proof is provided in the “Supplementary Materials”. $\square$

Following the establishment of NEDBU, it becomes imperative to ensure the non-negativity of players’ pollution emissions. This non-negativity is essential for maintaining the physical realism and validity of the model. To this end, we introduce certain constraints on the model parameters. These constraints not only guarantee the non-negativity of the emission strategies but also ensure the existence of a NEDBU. The subsequent proposition delineates these sufficient conditions.

### Proposition 5

To ensure that agents’ pollution emissions remain non-negative and that a NEDBU exists, the following conditions must simultaneously hold:

1. The parameter $\gamma$ must satisfy the range $0 \le \gamma \le 2$.
2. Players’ parameters $a_i$ must satisfy the following inequality:
  20 $$\begin{aligned} {\underline{a}} \ge \frac{\gamma N {\overline{a}}}{2 - \gamma + \gamma N}, \end{aligned}$$
  where $${\underline{a}}=\min \limits _{i \in \{1,2,..,N\}} a_i$$, $${\overline{a}}=\max \limits _{i \in \{1,2,..,N\}} a_i$$.
3. Players’ parameters $b_i$ must satisfy the following inequality:
  21 $$\begin{aligned} {\underline{b}} \ge \frac{\gamma N {\overline{b}}}{2 - \gamma + \gamma N}, \end{aligned}$$
  where $${\underline{b}}=\min \limits _{i \in \{1,2,..,N\}} b_i$$, $${\overline{b}}=\max \limits _{i \in \{1,2,..,N\}} b_i$$.
4. Players’ pollution emission capacity $a_i$ must be greater than or equal to the cost associated with pollution control $b_i$, meaning $a_i \ge b_i$, $\forall i \in \{1,2,..,N\}$.
5. $2 \delta \rho < 1$, implying that the effects of long-term benefits are relatively small and the environment has a high self-purification capacity, necessitating a demand for higher environmental quality.

### Proof

The detailed proof is provided in the “Supplementary Materials”. $\square$

Integrating the NEDBU (19) into the state dynamics and considering the signal $x_t$ as the realization of ${\widetilde{\eta }}$ at each time, we derive the real trajectory $\overline{S}$. This trajectory is represented as:

$$\begin{aligned} \overline{S}_{t+1} = x_t\left( \sum _{i=1}^{N} u_{i,t}^* + \delta \overline{S}_t\right) ,\quad \overline{S}_0 = S_0. \end{aligned}$$

The real trajectory $\overline{S}$ reflects the actual evolution of the pollutant stock over time. It incorporates the realized values of external factors ($x_t$), as well as the NEDBU of the players.

To ascertain the predicted trajectory $\hat{S}$ for each fixed time $t \in \{0,1,...,\infty \}$, and in the context of the truncated subgame $\Gamma (S, t, t+\overline{T})$, we obtain:

$$\begin{aligned} \hat{S}_{k+1}^t ={\overline{\eta }}_t\left( \sum _{i=1}^{N} \widetilde{u}_{i,k}^{t,*} + \delta \hat{S}_k^t\right) ,\quad \hat{S}_t = {\overline{S}}_t, \end{aligned}$$

where $k \in \{t,t+1,...,t+\overline{T}\}$, and ${\overline{S}}_t$ represents the real trajectory at time $t$.

The predicted trajectory $\hat{S}$ is based on the estimated values of external factors and the generalized NEDBU. It represents the players’ projection of how the pollutant stock might evolve, considering their expectations and the dynamic Bayesian updating of environmental uncertainties.

## Simulation results

In the context of this study, we detail the outcomes from our computational simulations that serve to validate the efficacy of our dynamic Bayesian learning methodology within the scope of ecological uncertainty. The generated signals adhere to a normal distribution, characterized by a mean of 0.5 and a variance of 1, thereby establishing the genuine mean value of the unknown parameter for the stochastic variable at 0.5. We have configured the model’s parameters such that $a_i = 8$ and $b_i = 3$ for each *i* ranging from 1 to *N*, with *N* being set to 5, and we have selected a decay factor, $\delta$, of 0.6.We considered the simulation results of the first 100 stages.

**Figure 2:**
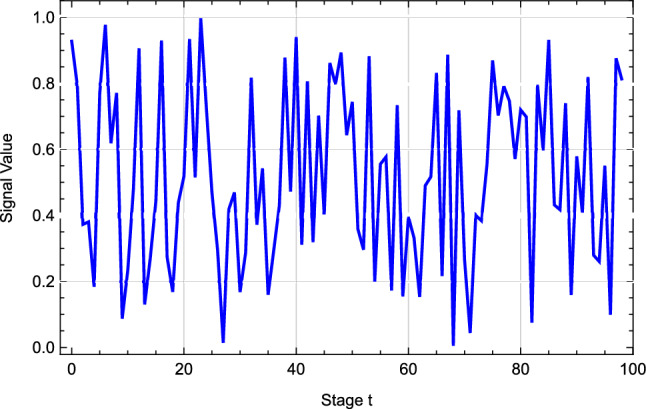
Received ecological uncertainty signals over time.

Figure 2 assumes that the signals follow a normal distribution. These signals are crucial inputs for the decision-making process of the players, reflecting the impact of environmental uncertainty on pollution control and the player’s estimations of unknown parameters based on updated signals.

**Figure 3:**
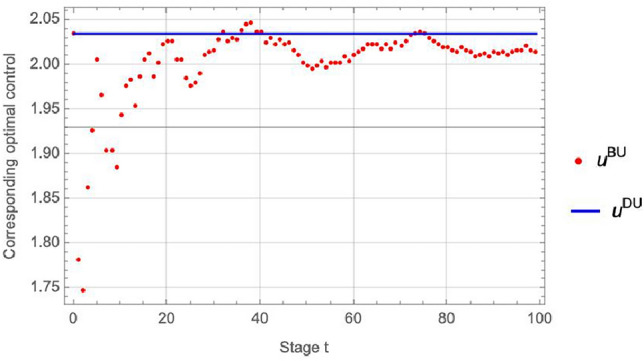
Comparative analysis of Nash equilibrium strategies: dynamic Bayesian updating vs. dynamic updating.

Figure 3 displays the player’s optimal strategies under varying scenarios, with red dots indicating strategies adopted under both parameter and structural uncertainties in the pollution control model, and the blue line representing strategies when only structural uncertainty exists, with known model parameters. As time progresses and the player continually updates their beliefs with information about the unknown parameters, we observe a gradual convergence of the NEDBU towards the blue line. This convergence highlights the dynamic Bayesian updating’s role in enhancing decision-making in pollution control.

**Figure 4:**
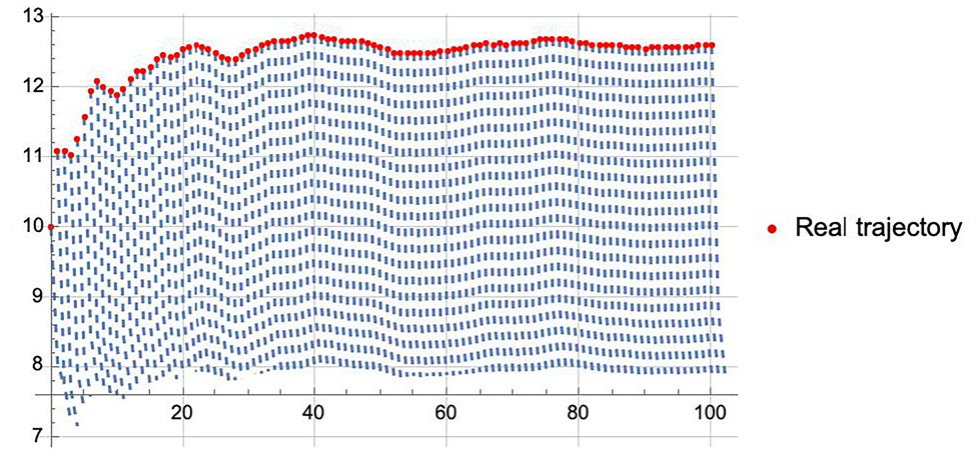
Delineation of trajectories: realized vs. predicted.

In Fig. 4, the red dots represent the real trajectory, indicating the pollution emission levels at each moment, under the condition that the model’s unknown parameters are set to their true values and the player implements the NEDBU. The blue line depicts the predicted trajectory, which is determined within each truncated subgame by the player’s generalized NEDBU, assuming an information horizon of 2. The predicted trajectory thus begins from the initial state $S_t$ and evolves according to the player’s strategies as determined by the generalized NEDBU in this subgame, leading to the blue line depicting the predicted trajectory.

**Figure 5:**
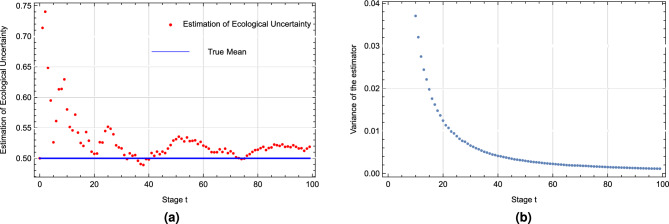
Evolving dynamics and variance analysis of estimators in predictive modeling.

Figure 5a illustrates the dynamic convergence of the players’ beliefs regarding the unknown parameter over time. It features two lines: the blue line represents the true value of the unknown parameter in the model at each moment, which is 0.5, and the red dots denote the players’ prior estimates of the unknown parameter at each time step, derived from the signals received. As time progresses, it is observed that these estimates gradually converge towards the true value.

Figure 5b depicts the variance of the players’ estimates of the unknown parameter relative to its true value at each moment. It is observed that as time progresses, this variance gradually approaches zero, indirectly indicating that our estimates become increasingly accurate.

The player’s optimal controls are influenced by their estimates of unknown parameters, which in turn are shaped by the signals received. To explore how signal inputs affect the NEDBU framework, we have generated three distinct sets of signals.

**Figure 6:**
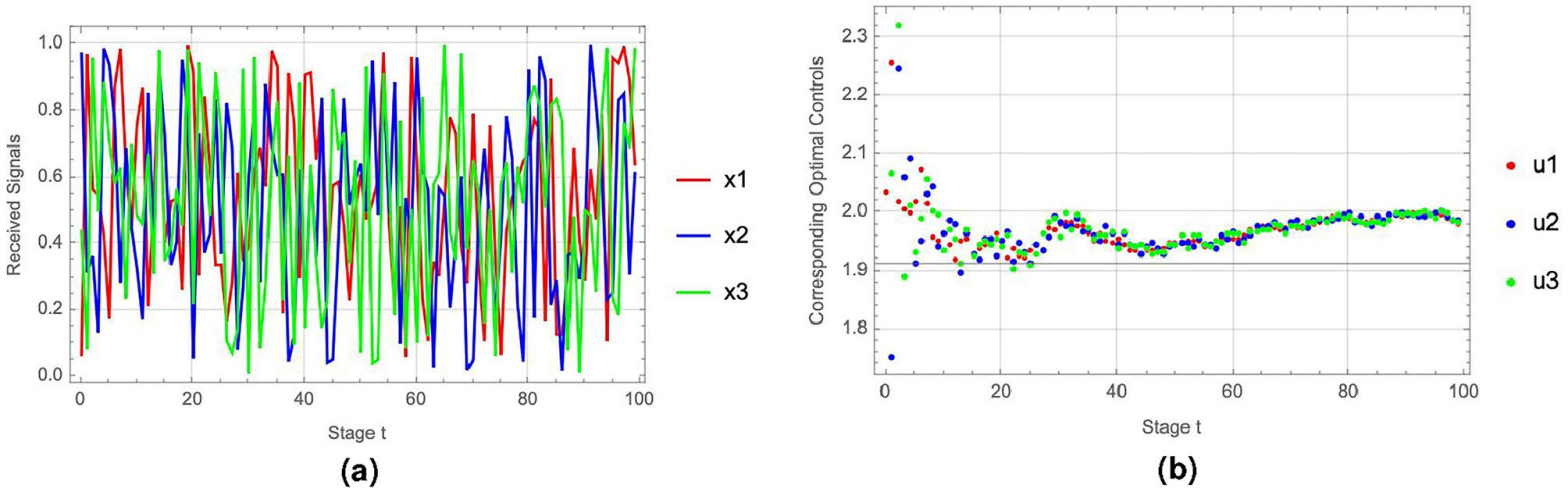
Nash equilibrium responses to varied signal groups under dynamic Bayesian updating.

In Fig. 6a, we present three sets of distinct signals to investigate their impact on the player’s pollution emission levels. Figure 6b illustrates the player’s NEDBU in response to these different signals, demonstrating how varied signal inputs lead to differing optimal control strategies by the player.

## Conclusions and future directions

This study delved into the dynamics of game theory under uncertainty, applying dynamic Bayesian updating to model the evolution of beliefs about unknown parameters. A pivotal finding is the compatibility of prior and posterior distributions across stages, confirming the convergence of beliefs toward the true values of the parameters. This outcome not only validates the robustness of our methodological construct but also illustrates the power of Bayesian updating in achieving optimal control and predicting accurate system trajectories. Through numerical simulations, we have discerned that the Nash equilibrium, informed by dynamic Bayesian updating, asymptotically aligns with the equilibrium derived from dynamic updating, as the beliefs about unknown parameters gradually converge to their true values. In the pollution control game, our findings indicate that players tend to emit less pollution when there is uncertainty in the model. This cautious behavior is due to the players’ tendency to be more conservative in their actions when faced with unknown factors. As such, we recommend that policymakers and stakeholders adopt a more cautious approach in scenarios where uncertainty exists, as this can lead to more prudent and environmentally beneficial outcomes.

The scope of this paper was confined to scenarios where unknown parameters are present only in the state equations. The extension of our approach to cases where these parameters also influence the payoff function remains unexplored. Future research could enhance model realism by: using an exponential distribution for the rate parameter in the fish war game, a gamma distribution for unknown information interval lengths, and a beta distribution for the depreciation rate in the investment in public goods game. Future work will also venture into the realm of differential games with uncertainty, scrutinizing the Bayesian learning process and its efficacy compared to game models with complete information. A critical consideration will be the application of Bayesian updating in continuous-time settings, which is a natural progression for differential games. This forthcoming research aims to bridge the gap in knowledge and further the application of machine learning in environmental and disaster studies.

## Supplementary Information

Supplementary Information.
