# Supplementary material for: Enhancing ecological uncertainty predictions in pollution control games through dynamic Bayesian updating
Source: Sci Rep. 2024 Jun 1;14:12594. doi: 10.1038/s41598-024-63234-1 (PMC11711505; doi:10.1038/s41598-024-63234-1)
Supplement: Supplementary file 1 — Supplementary Information. [file 41598_2024_63234_MOESM1_ESM.pdf]

Supplementary Materials

Jiangjing Zhou, Ovanes Petrosian, Hongwei Gao

1 Proof of Theorem 1

For any fixed time $t \in \{0, 1, ..., \infty\}$, the game in (4) and (6) is an N-person multistage game where $k \in \{t, t+1, ..., t+\overline{T}\}$. We first consider the last stage, that is, stage $t+\overline{T}$, and let the set containing all possible values of states be denoted by $S'_{t+\overline{T}}$. One has a single-stage game in which player i maximizes

$$\rho^{t+\overline{T}} g^i[t+\overline{T}, u^t_{t+\overline{T}}, S^t_{t+\overline{T}},], \quad for \quad i = 1, 2, ..., N, \tag{22}$$

where $u^t_{t+\overline{T}} = (u^t_{1,t+\overline{T}}, ..., u^t_{N,t+\overline{T}})$ and $S^t_{t+\overline{T}} = S \in S'_{t+\overline{T}}$.

The solution to the problem in (22) is the Nash equilibrium of an N-person static game, which yields a set of strategies $\{\widetilde{u}^{t,*}_{i,t+\overline{T}}(S, \xi_t(\theta))$, for $i = 1, 2, ..., N\}$. Substituting the game equilibrium strategies into agent $i's$ objective yields his game equilibrium payoff in stage $t+\overline{T}$

$$V^t_i(t+\overline{T}, S, \xi_t(\theta)) = \rho^{t+\overline{T}} g^i[t+\overline{T}, \widetilde{u}^{t,*}_{t+\overline{T}}, S] + V^t_i(t+\overline{T}+1, f(E(\widetilde{\eta}|\overline{\theta}_t), \widetilde{u}^{t,*}_{t+\overline{T}}, S), \xi_t(\theta)).$$

Using the relation for $k = t+\overline{T}$ in (7) and $V^t_i(t+\overline{T}+1, \cdot) = 0$, we have the problem

$$V^t_i(t+\overline{T}, S, \xi_t(\theta)) = \max_{u^t_{i,t+\overline{T}}} \left\{ \rho^{t+\overline{T}} g^i[t+\overline{T}, u^t_{t+\overline{T}}, S] \right\}, \quad for \quad i = 1, 2, ..., N, \tag{23}$$

with $S^t_{t+\overline{T}} = S \in S'_{t+\overline{T}}$.

The problem in (23) is equivalent to the problem in (22).

Now consider the problem in stage $t+\overline{T}-1$. The problem becomes a game in which agent i

$$\max_{u^t_{i,t+\overline{T}-1}, u^t_{i,t+\overline{T}}} \left\{ \sum_{\zeta=t+\overline{T}-1}^{t+\overline{T}} \rho^\zeta g^i[\zeta, u^t_\zeta, S^t_\zeta] \right\}, \quad for \quad i = 1, 2, ..., N,$$

subject to

$$S_{t+\overline{T}}^{t} = f(E(\widetilde{\eta}|\overline{\theta}_t), u_{t+\overline{T}-1}^{t}, S_{t+\overline{T}-1}^{t}), \quad S_{t+\overline{T}-1}^{t} \in S_{t+\overline{T}-1}'.$$

Using the analysis in stage $t+\overline{T}$, the stage $t+\overline{T}-1$ problem can be expressed as a single-stage game in which agent i seeks to maximize

$$\begin{aligned} &\rho^{t+\overline{T}-1} g^i[t+\overline{T}-1, u_{t+\overline{T}-1}^{t}, S_{t+\overline{T}-1}^{t}] + V_i^t(t+\overline{T}, S_{t+\overline{T}}^{t}, \xi_t(\theta)) \\ &= \rho^{t+\overline{T}-1} g^i[t+\overline{T}-1, u_{t+\overline{T}-1}^{t}, S_{t+\overline{T}-1}^{t}] + V_i^t(t+\overline{T}, f(E(\widetilde{\eta}|\overline{\theta}_t), u_{t+\overline{T}-1}^{t}, S_{t+\overline{T}-1}^{t}), \xi_t(\theta)). \end{aligned} \tag{24}$$

The game equilibrium for (24) can be expressed as a set of strategies $\{\widetilde{u}_{i,t+\overline{T}-1}^{t,*}(S, \xi_t(\theta))\}_{i=1,2,\ldots,N}$ for its explicit dependence on $S_{t+\overline{T}-1}^{t} \in S_{t+\overline{T}-1}'$. Note that the relation for $k = t+\overline{T}-1$ in (7) characterizes the game equilibrium of (24).

Proceeding recursively onward for stage $k \in \{t+\overline{T}-2, t+\overline{T}-3, ..., 0\}$, the stage k problem can be expressed as a single-stage game in which agent i seeks to maximize

$$\rho^k g^i[k, u_k^t, S] + V_i^t(k+1, f(E(\widetilde{\eta}|\overline{\theta}_t), u_k^t, S), \xi_t(\theta)), \quad for \quad i = 1, 2, ..., N, \tag{25}$$

where $S_k^t = S \in S_k'$.

The game equilibrium for the problem in (25) can be expressed as a set of strategies $\{\widetilde{u}_{i,k}^{t,*}(S, \xi_t(\theta))\}$ because of its explicit dependence on S. Note that the relation for $k \in \{0, 1, 2, ..., t+\overline{T}-2\}$ in (7) characterizes the game equilibrium of (25).

Hence Theorem 1 follows.

2 Proof of Theorem 2

In our case, X_1, X_2, ... is an infinite sequence of independent and identically distributed (i.i.d.) Lebesgue integrable random variables with expected value $E(X_1) = E(X_2) = ... = \mu$. As a result, we can obtain

$$\begin{aligned} E(M_n) &= \frac{\kappa_0\mu_0}{\kappa_0+n} + \frac{1}{\kappa_0+n}(EX_0 + EX_1 + \cdots + EX_{n-1}) \\ &= \frac{\kappa_0\mu_0}{\kappa_0+n} + \frac{n\mu}{\kappa_0+n} \\ &= \frac{\kappa_0\mu_0 + n\mu}{\kappa_0+n} = \frac{\frac{\kappa_0\mu_0}{n} + \mu}{\frac{\kappa_0}{n} + 1}. \end{aligned} \tag{26}$$

If we consider the limit of (26) with respect to n, then

$$\lim_{n\to\infty} E(M_n) = \frac{0+\mu}{0+1} = \mu.$$

3 Proof of Theorem 3

Using Eq. (16), we can obtain

$$\begin{aligned}
\lim_{n\to\infty} E\left(\frac{B_n}{\kappa_n\left(\alpha_n-1\right)}\right) &= \lim_{n\to\infty} E\left(\frac{\beta_0+\sum_{m=0}^{n-1} Y_m}{\left(\kappa_0+n\right)\left(\alpha_0+\frac{1}{2}n-1\right)}\right) \\
&= \lim_{n\to\infty}\left(\frac{\beta_0}{\left(\kappa_0+n\right)\left(\alpha_0+\frac{1}{2}n-1\right)}+\frac{E\left(\sum_{m=0}^{n-1} Y_m\right)}{\left(\kappa_0+n\right)\left(\alpha_0+\frac{1}{2}n-1\right)}\right) \\
&= \lim_{n\to\infty}\frac{\beta_0}{\left(\kappa_0+n\right)\left(\alpha_0+\frac{1}{2}n-1\right)}+\lim_{n\to\infty}\frac{E\left(\sum_{m=0}^{n-1} Y_m\right)}{\left(\kappa_0+n\right)\left(\alpha_0+\frac{1}{2}n-1\right)} \\
&= \lim_{n\to\infty}\frac{\sum_{m=0}^{n-1} E\left(Y_m\right)}{\left(\kappa_0+n\right)\left(\alpha_0+\frac{1}{2}n-1\right)},
\end{aligned} \tag{27}$$

the last line of Eq. (27) holds since the random variables $Y_0, Y_1, ..., Y_{n-1}$ are independent. This independence permits the interchange of the summation and expectation operators in the concluding equation.

To address the series of EY_m, we aim to demonstrate that $\lim_{m\to\infty} E\left(Y_m\right)=d$, where d is a specified constant. Consequently, we proceed to construct

$$\begin{aligned}
EY_m &= \frac{\kappa_m}{2\left(\kappa_m+1\right)} E\left(X_m-M_m\right)^2 \\
&= \frac{\kappa_m}{2\left(\kappa_m+1\right)}\left[D\left(X_m-M_m\right)+\left(E\left(X_m-M_m\right)\right)^2\right] \\
&= \frac{\kappa_m}{2\left(\kappa_m+1\right)}\left[DX_m-DM_m+\left(E\left(X_m-M_m\right)\right)^2\right],
\end{aligned} \tag{28}$$

where M_m is the corresponding random variable that represents the belief μ_m at time m.

In the context of addressing equation (28), it becomes imperative to evaluate DM_m and $E(X_m - M_m)$. Our focus will be on delineating DM_m, drawing upon the framework provided by equation (16).

$$\begin{aligned}
DM_m &= \frac{1}{\left(\kappa_0+m\right)^2} D(\kappa_0\mu_0+X_0+X_1+...+X_{m-1}) \\
&= \frac{mDX}{\left(\kappa_0+m\right)^2}.
\end{aligned} \tag{29}$$

The final line of (29) is derived because $X_0, X_1, ..., X_{m-1}$ are independent and identically distributed

random variables. Consequently, it is permissible to posit that the expected value $E(X_t) = c = \mu$ and the variance $D(X_t) = 2d = 1/\lambda$, where $c, d > 0$ and $t = 0, 1, 2, \ldots, +\infty$.

Substituting M_n in (16) into $E(X_m - M_m)$, we can derive

$$\begin{aligned} E\left(X_m - M_m\right) &= EX_m - \frac{\kappa_0\mu_0 + mEX_m}{\kappa_0 + m} \\ &= c - \frac{\kappa_0\mu_0 + mc}{\kappa_0 + m} \\ &= \frac{\kappa_0(c - \mu_0)}{\kappa_0 + m}. \end{aligned}$$

Therefore,

$$\left(E\left(X_m - M_m\right)\right)^2 = \frac{\kappa_0^2\left(c - \mu_0\right)^2}{\left(\kappa_0 + m\right)^2}. \tag{30}$$

By integrating equation (30) into equation (28), we are able to derive

$$\begin{aligned} EY_m &= \frac{\kappa_m}{2\left(\kappa_m + 1\right)}\left(DX - \frac{mDX}{\left(\kappa_0 + m\right)^2} + \frac{\kappa_0^2\left(c - \mu_0\right)^2}{\left(\kappa_0 + m\right)^2}\right) \\ &= \frac{\kappa_0 + m}{2\left(\kappa_0 + m + 1\right)}DX - \frac{m}{2\left(\kappa_0 + m + 1\right)\left(\kappa_0 + m\right)}DX + \frac{\kappa_0^2\left(c - \mu_0\right)^2}{2\left(\kappa_0 + m + 1\right)\left(\kappa_0 + m\right)}. \end{aligned} \tag{31}$$

Contemplating the limit as m approaches infinity in equation (31), we arrive at

$$\begin{aligned} \lim_{m\to\infty} EY_m = \lim_{m\to\infty} &\frac{\frac{\kappa}{m} + 1}{2\left(\frac{\kappa_0}{m} + 1 + \frac{1}{m}\right)}DX - \lim_{m\to\infty} \frac{1}{2\left(\frac{\kappa_0}{m} + 1\right)\left(\kappa_0 + m + 1\right)} \\ &+ \lim_{m\to\infty} \frac{\kappa_0^2\left(c - \mu_0\right)^2}{2\left(\kappa_0 + m + 1\right)\left(\kappa_0 + m\right)} \\ &= \frac{1}{2}DX = d > 0. \end{aligned}$$

Employing the formalism of the limit definition, it is established that for every $\epsilon > 0$, there exists a corresponding N_ϵ such that for all $n > N_\epsilon$, the inequality $|EY_n - d| < \epsilon$ holds true. This can equivalently be articulated as $d - \epsilon < EY_n < d + \epsilon$.

And then we are positioned to proceed with the demonstration that

$$\lim_{n\to\infty} \frac{\sum_{m=0}^{n-1} E(Y_m)}{\left(\kappa_0 + n\right)\left(\alpha_0 + \frac{1}{2}n - 1\right)} = 0.$$

Given that $\lim n \to \infty \, (\kappa 0 + n) \left(\alpha_0 + \frac{1}{2}n - 1\right) \neq 0$, it logically follows that

$$\begin{aligned}
\lim_{n\to\infty} \frac{\sum_{m=0}^{n-1} E(Y_m)}{(\kappa_0 + n)\left(\alpha_0 + \frac{1}{2}n - 1\right)} &= \frac{\lim_{n\to\infty} \sum_{m=0}^{n-1} E(Y_m)}{\lim_{n\to\infty} (\kappa_0 + n)\left(\alpha_0 + \frac{1}{2}n - 1\right)} \\
&= \frac{\sum_{m=0}^{\infty} E(Y_m)}{\lim_{n\to\infty} (\kappa_0 + n)\left(\alpha_0 + \frac{1}{2}n - 1\right)} \\
&= \frac{\sum_{m=0}^{N_\epsilon} E(Y_m) + \sum_{m=N_\epsilon+1}^{\infty} E(Y_m)}{\lim_{n\to\infty} (\kappa_0 + n)\left(\alpha_0 + \frac{1}{2}n - 1\right)} \\
&= \frac{\sum_{m=0}^{N_\epsilon} E(Y_m)}{\lim_{n\to\infty} (\kappa_0 + n)\left(\alpha_0 + \frac{1}{2}n - 1\right)} + \frac{\sum_{m=N_\epsilon+1}^{\infty} E(Y_m)}{\lim_{n\to\infty} (\kappa_0 + n)\left(\alpha_0 + \frac{1}{2}n - 1\right)} \\
&= \frac{\sum_{m=N_\epsilon+1}^{\infty} E(Y_m)}{\lim_{n\to\infty} (\kappa_0 + n)\left(\alpha_0 + \frac{1}{2}n - 1\right)}.
\end{aligned} \tag{32}$$

The derivation of the final line in equation (32) is predicated on the fact that the series $\sum_{m=0}^{N_\epsilon} E(Y_m)$ converges to a finite value, i.e., its sum is less than infinity.

Starting from the definition of the limit of a sequence, $\lim_{m\to\infty} E(Y_m) = d$, it follows that

$$\sum_{m=N_\epsilon+1}^{\infty} (d - \epsilon) < \sum_{m=N_\epsilon+1}^{\infty} E(Y_m) < \sum_{m=N_\epsilon+1}^{\infty} (d + \epsilon). \tag{33}$$

The left-hand side of inequality (33) can be reformulated as

$$\sum_{m=N_\epsilon+1}^{\infty} (d - \epsilon) = \lim_{n\to\infty} \sum_{m=N_\epsilon+1}^{n} (d - \epsilon) = \lim_{n\to\infty} (n - N_\epsilon - 1)(d - \epsilon). \tag{34}$$

By integrating the expressions provided in equations (32), (33), and (34), we are able to deduce the subsequent conclusion:

$$\begin{aligned}
\frac{\lim_{n\to\infty} (n - N_\epsilon - 1)(d - \epsilon)}{\lim_{n\to\infty} (\kappa_0 + n)\left(\alpha_0 + \frac{1}{2}n - 1\right)} &< \frac{\sum_{m=N_\epsilon+1}^{\infty} E(Y_m)}{\lim_{n\to\infty} (\kappa_0 + n)\left(\alpha_0 + \frac{1}{2}n - 1\right)} \\
&< \frac{\lim_{n\to\infty} (n - N_\epsilon - 1)(d + \epsilon)}{\lim_{n\to\infty} (\kappa_0 + n)\left(\alpha_0 + \frac{1}{2}n - 1\right)},
\end{aligned} \tag{35}$$

the leftmost term of the inequality (35) is

$$\begin{aligned}
\frac{\lim_{n\to\infty} (n - N_\epsilon - 1)(d - \epsilon)}{\lim_{n\to\infty} (\kappa_0 + n)\left(\alpha_0 + \frac{1}{2}n - 1\right)} &= \lim_{n\to\infty} \frac{(n - N_\epsilon - 1)(d - \epsilon)}{(\kappa_0 + n)\left(\alpha_0 + \frac{1}{2}n - 1\right)} \\
&= \lim_{n\to\infty} \frac{(1 - \frac{N_\epsilon + 1}{n})(d - \epsilon)}{(\frac{\kappa_0}{n} + 1)(\alpha_0 + \frac{1}{2}n - 1)} \\
&= \frac{d - \epsilon}{\infty} \\
&= 0.
\end{aligned}$$

Similarly, identifying the rightmost side of (35) as 0, and applying the principles of the squeeze theorem, we arrive at the following conclusion:

$$\lim_{n\to\infty} \frac{\sum_{m=0}^{n-1} E(Y_m)}{(\kappa_0 + n)\left(\alpha_0 + \frac{1}{2}n - 1\right)} = 0.$$

Independent of the initial parameter selections for α_0, β_0, μ_0, and κ_0, as t approaches infinity, it is feasible to assert that the variance associated with the estimate of the unknown mean converges towards zero.

4 Proof of Proposition 4

Denote by $V_i^t(k, S, \mu_t, k_t, \alpha_t, \beta_t)$ the value function of player i. The Hamilton–Jacobi–Bellman (HJB) equation is given by

$$V_i^t(k, S, \mu_t, k_t, \alpha_t, \beta_t) =$$
$$\max_{u_{i,k}^t} \left\{ \rho^k (u_{i,k}^t (a_i - u_{i,k}^t - \gamma \sum_{j\neq i}^{N} u_{j,k}^t) - b_i S) + V_i^t(k+1, \overline{\eta}_t (\sum_{i=1}^{N} u_{i,k}^t + \delta S), \mu_t, k_t, \alpha_t, \beta_t) \right\}.$$

We first consider the linear-state structure of our model and hypothesize that the value function is linear:

$$V_i^t(k, S, \mu_t, k_t, \alpha_t, \beta_t) = A_i^t(k, \mu_t, k_t, \alpha_t, \beta_t) S + B_i^t(k, \mu_t, k_t, \alpha_t, \beta_t),$$

for $k = t, t+1, ..., t+\overline{T}$ and $i = 1, 2, ..., N$.

Using the Hamilton–Jacobi–Bellman (HJB) equation and first-order conditions, we find that

$$\rho^k (a_i - 2u_{i,k}^t - \gamma \sum_{j\neq i}^{N} \widetilde{u}_{j,k}^{t,*}) + \overline{\eta}_t \frac{\partial V_i^t}{\partial S_{k+1}} = 0.$$

Equating the coefficients of S, we derive the coefficients of the value function:

$$A_i^t(k, \mu_t, k_t, \alpha_t, \beta_t) = \frac{b_i((\overline{\eta}_t \delta)^{t+\overline{T}+1-k} \rho^{t+\overline{T}+1} - \rho^k)}{1 - \overline{\eta}_t \delta \rho}.$$

Building upon our earlier analysis, we define the generalized Nash equilibrium strategies with dynamic Bayesian updating for each player $i = 1, 2, ..., N$ in the subgame $\Gamma(S, t, t+\overline{T})$. These strategies are articulated as follows:

$$\widetilde{u}_{i,k}^{t,*}(S, \mu_t, k_t, \alpha_t, \beta_t) = \frac{a_i}{2-\gamma} - \frac{\gamma a}{(2-\gamma+\gamma N)(2-\gamma)}$$
$$+ \frac{\overline{\eta}_t}{\rho^k (2-\gamma)(1-\overline{\eta}_t \delta \rho)} \left(b_i - \frac{\gamma b}{2-\gamma+\gamma N} \right) \left((\overline{\eta}_t \delta)^{t+\overline{T}-k} \rho^{t+\overline{T}+1} - \rho^{k+1} \right),$$

where $\overline{\eta}_t = E(\widetilde{\eta}|\mu_t, \alpha_t/\beta_t)$, $a = \sum_{i=1}^N a_i$, $b = \sum_{i=1}^N b_i$, and $k \in \{t, t+1, t+2, ..., t+\overline{T}\}$, for $t \in \{0, 1, ..., \infty\}$.

Applying Definition 2, the Nash Equilibrium with dynamic Bayesian updating for the game $\Gamma(S_0, t_0, \infty)$ can be readily verified. These strategies are directly derived from the generalized Nash equilibrium strategies, demonstrating the intricate dynamics of the game under the influence of Bayesian updating.

5 Proof of Proposition 5

We commence by establishing the non-negativity of the initial and subsequent terms within equation (19). The exposition unfolds as follows:

$$\begin{aligned} \frac{a_i}{2-\gamma} - \frac{\gamma a}{(2-\gamma+\gamma N)(2-\gamma)} &\geq \frac{a_i}{2-\gamma} - \frac{\gamma N \overline{a}}{(2-\gamma+\gamma N)(2-\gamma)}, \\ &\geq \frac{\underline{a}}{2-\gamma} - \frac{\gamma N \overline{a}}{(2-\gamma+\gamma N)(2-\gamma)}. \end{aligned} \tag{36}$$

Upon substitution of condition (20) into equation (36), we deduce that:

$$\frac{a_i}{2-\gamma} - \frac{\gamma a}{(2-\gamma+\gamma N)(2-\gamma)} \geq 0. \tag{37}$$

A parallel argument, incorporating condition (21), yields:

$$\frac{b_i}{2-\gamma} - \frac{\gamma b}{(2-\gamma+\gamma N)(2-\gamma)} \geq 0. \tag{38}$$

Our subsequent objective is to validate that:

$$\frac{\overline{\eta}_t \rho}{(1-\overline{\eta}_t \delta\rho)}(1-(\overline{\eta}_t \delta\rho)^{\overline{T}}) < 1. \tag{39}$$

This is equivalently demonstrated by showing:

$$\overline{\eta}_t \rho(1-(\overline{\eta}_t \delta\rho)^{\overline{T}}) < (1-\overline{\eta}_t \delta\rho). \tag{40}$$

Given that:

$$\overline{\eta}_t \rho\delta(1-(\overline{\eta}_t \delta\rho)^{\overline{T}}) < \overline{\eta}_t \rho(1-(\overline{\eta}_t \delta\rho)^{\overline{T}}), \tag{41}$$

it suffices to establish that:

$$\overline{\eta}_t \rho\delta(1-(\overline{\eta}_t \delta\rho)^{\overline{T}}) < 1-\overline{\eta}_t \delta\rho. \tag{42}$$

For expediency, we denote $\overline{\eta}_t \delta\rho$ as $0 < r_t < 1$. Hence, we reformulate equation (42) to:

$$(r_t)^{\overline{T}} > 1 - \frac{(1 - r_t)}{r_t} = \frac{2r_t - 1}{r_t}. \tag{43}$$

Acknowledging that $\overline{\eta}_t < 1 < \frac{1}{2\delta\rho}$ in the fifth condition, we ascertain:

$$2r_t - 1 < 0.$$

Thus, it logically follows:

$$(r_t)^{\overline{T}} > 0 > \frac{2r_t - 1}{r_t},$$

which affirms the veracity of (43).

Consequently, we substantiate that the Nash equilibrium with dynamic Bayesian updating, as explicated by equation (19), remains non-negative.
